# Regulation of ofloxacin resistance in *Escherichia coli* strains causing calf diarrhea by quorum-sensing acyl-homoserine lactone signaling molecules

**DOI:** 10.3389/fvets.2025.1540132

**Published:** 2025-02-05

**Authors:** Zi Wang, Miao Sun, Yongqiang Wang, Jinchuan Shi, Wei Gao, Dongxu Han, Fanjun Zeng, Sanren, Liyin Du, Hongxia Ma, Kai Liu

**Affiliations:** ^1^College of Animal Science and Technology, Inner Mongolia Minzu University, Tongliao, China; ^2^Inner Mongolia Engineering Technology Research Center for Prevention and Control of Beef Cattle Diseases, Tongliao, China; ^3^Beef Cattle Industry School of Inner Mongolia Autonomous Region, Tongliao, China; ^4^Zhalantun Vocational College, Hulunbeier, China; ^5^Tongliao Agricultural and Animal Product Quality Safety Center, Tongliao, China; ^6^College of Animal Science and Technology, Jilin Agricultural University, Changchun, China

**Keywords:** *Escherichia coli*, drug resistance, quorum-sensing molecule, transcriptomic sequencing, differentially expressed genes

## Abstract

*Escherichia coli* is a major pathogen responsible for calf diarrhea. However, it has developed resistance to many antimicrobial drugs for their inappropriate usage. The bacterial quorum sensing system transmits information between bacteria, it's important in regulating bacterial virulence, drug and acid resistance and so on. This system can found in Gram-negative bacteria and operates through acyl-homoserine lactone (AHL) signaling molecules. In this study, a type I quorum sensing AHL, N-Octanoyl-L-Homoserine lactone (C8), was added to *E. coli* growth medium to investigate its regulatory functions in drug resistance. After screening out the strains of *E. coli* that showed an obvious regulatory effect to the drug ofloxacin (OFX), transcriptomic sequencing was performed on the *E. coli* strains from the sub-inhibitory concentration group that concentration plus C8 group, and the control group. It shows that C8 significantly influenced resistance to OFX and the minimum inhibitory concentration of OFX in the tested strain was significantly increased. To Analyze transcriptome sequencing results identified 415 differentially expressed genes between the control and sub-inhibitory concentration groups, of which 201 were up-regulated and 214 were down. There were 125 differentially expressed genes between bacteria treated with a sub-inhibitory concentration of OFX and those treated with C8, of which 102 were up-regulated and 23 were down. Finally, It found that to adding the C8 significantly increased the resistance of tested bacteria to OFX. Data from transcriptome sequencing on differently expressed genes helps to explain how the type I quorum sensing system controls drug resistance in *E. coli*.

## 1 Introduction

*Escherichia coli* is a widely distributed Gram-negative bacterium and is the most numerous facultative anaerobe inhabiting the intestines of warm-blooded animals (1, 2). Many *E. coli* strains are harmless or even beneficial to the host; however, some strains can be pathogenic to both humans and animals (3). At present, one of the more challenging diseases facing veterinarians and farmers today is colibacillosis, which is highly contagious and can cause severe diarrhea and septicemia in calves, with especially high morbidity and mortality rates in younger animals, resulting in significant financial loss to farmers and the cattle industry (4, 5).

It is common practice in the livestock industry to routinely administer large amounts of antimicrobials to prevent and treat infectious diseases, as well as to promote growth (6, 7). While antimicrobials are important in the prevention and treatment of bacterial infections, their misuse has led to increased drug resistance and the development of multidrug-resistant strains (8). This increased antimicrobial resistance (AMR) is becoming a concern for veterinarians and livestock producers (9). As previous studies show, cattle are an important source of AMR bacteria. The resistance in *E. coli* is especially concerning due to the risk it poses to human health via the food chain. *E. coli* strains are normally resident in the intestinal tract and bovine carriers are often asymptomatic. Consequently, the transfer of resistance from non-pathogenic to pathogenic strains can occur with ease in the same environment, resulting in the emergence of multidrug-resistant strains, as well as increasing the selection pressure for the clinical therapeutic use of antimicrobial drugs (10).

Bacterial cells can communicate with one another through the production and detection of signaling molecules, a mechanism known as quorum sensing (QS). This signaling is mediated by molecules known as autoinducers (AIs). Two types of bacterial cell-cell communication are recognized, namely, the AI-1 and AI-2 QS regulatory systems (11). The recent discovery of these microbial QS systems has provided new hope for investigating the regulatory mechanisms underlying the development of drug resistance and thus overcoming this resistance (12). It has been found that QS systems modulate various cellular processes in microorganisms, particularly in the regulation of drug efflux pumps and the formation of microbial biofilms (13, 14). The currently most studied QS pathways are mediated by acyl homoserine lactones (AHLs or AI-1) in Gram-negative bacteria (15). Bacteria produce and detect specific signaling molecules to coordinate their gene expression and physiological activities in relation to the bacterial cell density, promoting biofilm formation, the secretion of extracellular enzymes, synthesis of antimicrobial substance synthesis, competence, and virulence factor expression. This complex signaling network allows large populations of microbial cells to exhibit a multicellular behavioral pattern in response to changing environmental conditions (16–19).

This study aimed to investigate the impact of QS molecule C8 on the antibiotic resistance in *E. coli* strains that cause calf diarrhea to common antibiotics and to elucidate the underlying molecular mechanisms. In this study, OFX, enrofloxacin (ENR), doxycycline (DOX), florfenicol (FFC), and tetracycline (TET) were used to determine their minimum inhibitory concentration (MICs). In addition, 200 mg/L (1 × 10^−3^ mol/L) of the AHL, N-Octanoyl-L-Homoserine lactone (C8), a type I QS molecule, was added to the growth medium to identify drugs in which resistance was regulated by C8. The transcriptomes of three groups of bacteria, one treated with a sub-inhibitory drug concentration, another treated with a sub-inhibitory drug concentration plus C8, and a control group, were sequenced, and differentially expressed genes (DEGs) were identified. The expression of these genes was verified using RT-qPCR to determine the effects of C8 on drug-resistant *E. coli* causing diarrhea in calves. This study provides data supporting the research and development of new drugs and drug resistance inhibitors to counteract the development of bacterial resistance, thereby reducing the economic losses caused by *E. coli* diarrhea in calves.

## 2 Materials and methods

### 2.1 Bacterial strain

Calf diarrhea *E. coli* was isolated from a large-scale beef cattle farm in Tongliao City and kept in the Laboratory of Preventive Veterinary Medicine, College of Animal Science and Technology, Inner Mongolia MINZU University.

### 2.2 Drug sensitivity testing

The sensitivities of the bacterial isolate to five antimicrobial drugs, namely, OFX, ENR, DOX, FFC, and TET, were tested using the broth microdilution method, as described in the Clinical and Laboratory Standards Institute (CLSI) guidelines (20). Briefly, bacteria were grown in LB broth until reaching an OD600 of 0.5. Two hundred microliters of serial dilutions of the antimicrobial agents were placed in sterile 96-well polystyrene plates; the concentrations (wells 1–15) were 512, 256, 128, 64, 32, 16, 8, 4, 2, 1, 0.5, 0.25, 0.125, 0.0625, and 0.03125 μg/mL. Then, 100 μL of the bacterial suspension was added. The 16th well was used as a blank control (LB culture medium containing no antibiotics). Background blank and positive controls were included in each experiment. Experiments were performed with at least three independent replicates. The plates were incubated at 37°C for 24 h. The lowest concentration with no visible bacteria growth was considered the MIC (21, 22). The MIC of each antibiotic was classified as resistant (R), intermediate (I), or susceptible (S) based on CLSI breakpoints, or the National Antibiotic Resistance Monitoring System (NARMS) breakpoints for intestinal bacteria when the CLSI breakpoints were unavailable (23).

### 2.3 Effect of AHL signaling molecules on drug resistance in *E. coli*

As described by Zhang et al. (24), 40 mg/L (2 × 10^−4^ mol/L), 60 mg/L (3 × 10^−4^ mol/L), 80 mg/L (4 × 10^−4^ mol/L), 100 mg/L (5 × 10^−4^ mol/L), and 200 mg/L (1 × 10^−3^ mol/L) of the AHL C8 were added for pre-experimentation, with a concentration of 200 mg/L (1 × 10^−3^ mol/L) ultimately selected for the subsequent experiments. After the addition of the antibiotics and bacterial suspension described in 2.2, 200 mg/L (1 × 10^−3^ mol/L) C8 was added, and the plates were incubated at 37°C for 24 h. The MIC results were observed.

### 2.4 Total RNA extraction and transcriptomic sequencing

Three groups of bacterial cells were established, namely, a group treated with a sub-inhibitory concentration of OFX, a second group treated with a sub-inhibitory concentration of OFX plus C8, and a control group. Specifically, Group 1 (TL-3) was treated with 16 μg/ml OFX, Group 2 (TL-4) was treated with 64 μg/ml OFX with the addition of 200 mg/L (1 × 10^−3^ mol/L) C8, and the control group (TL-2) had no OFX and C8. The bacteria were collected for total RNA extraction when they reached the exponential growth phase (OD600 = 0.5). Total RNA was extracted from the TL-2, TL-3, and TL-4 groups using a Bacteria RNA Extraction Kit (Tiangen Biochemical Technology (Beijing) Co., Ltd.), according to the manufacturer's instructions. The RNA samples were sent to Guangzhou Gene Denovo Biotechnology Co. for high-throughput sequencing (Illumina's Novaseq 6000 platform) and transcriptomic analysis. The raw reads were filtered to remove low-quality reads, including reads containing adapters, reads containing nucleotides with quality scores lower than 20, and reads with unknown nucleotides larger than 10%, before mapping to the ribosomal RNA (rRNA) database using Bowtie 2 version 2.2.8. The rRNA-mapped reads were removed, and the remaining reads were mapped to the reference genome. The reference genome sequence accession number was CP103295-CP103297. The prediction of the new transcripts was conducted using Rockhopper. Gene expression levels were normalized using the Fragments Per Kilobase of transcript per Million mapped reads (FPKM) method. The edgeR package (version 3.12.1; http://www.r-project.org) was used to identify DEGs across samples or groups using the criteria of a fold change ≥2 and a false discovery rate (FDR) < 0.05.

### 2.5 Analysis of DGEs

All DEGs were annotated using the Gene Ontology (GO) and the Kyoto Encyclopedia of Genes and Genomes (KEGG) databases. GO functional annotation was performed to describe the molecular functions, cellular components, and biological processes associated with the DEGs. KEGG enrichment analysis was used to identify the metabolic and signaling pathways associated with the DGEs, with significant enrichment determined by comparison to the annotation of reference transcripts (FDR ≤ 0.05). In addition, 9 DEGs (Supplementary Table 1) were selected for verification of their expression levels using quantitative reverse transcription-polymerase chain reaction (qRT-PCR). All qPCR reactions were performed in a total volume of 20 μL. The cycling parameters included an initial denaturation at 95°C for 30 s, followed by 40 cycles of 95°C for 10 s, and finally 60°C for 30 s. The 16S gene was used as an internal control and the changes in relative gene expression were calculated using with the 2^−Δ*ΔCt*^ method.

### 2.6 Nucleotide sequence accession numbers

All sequences were deposited in the NCBI Sequence Read Archive and are publicly accessible under accession number PRJNA1163313. These files can be accessed via the following link: https://www.ncbi.nlm.nih.gov/bioproject/PRJNA1163313 (accessed on 20 September 2024).

### 2.7 Statistical analysis

Statistical analysis was performed using the GraphPad Prism 5 software package (Graph Software, San Diego, CA, USA). All data are expressed as the mean ± standard error based on three independent experiments. A *p*-value < 0.05 was considered statistically significant.

## 3 Results

### 3.1 Drug sensitivity results

The MIC values of the five drugs, ENR, OFX, DOX, FFC, and TET, after treatment with 200 mg/L (1 × 10^−3^ mol/L) C8 are shown in Table 1. In the C8-treated group, the MIC values for OFX were increased from an initial 32–128 μg/ml, while those for ENR were increased from an initial 256–512 μg/ml, and the values for DOX, FFC, and TET remain unchanged.

**Table 1:** Results of C8-induced drug resistance in *E. coli*.

	**Ofloxacin**	**Doxycycline**	**Enrofloxacin**	**Tetracycline**	**Florfenicol**
Control group	32/R	4/R	256/R	>512/R	>512/R
C8 treated group	128/R	4/R	>512/R	>512/R	>512/R

### 3.2 Distribution statistics of gene expression value

**Figure 1:**
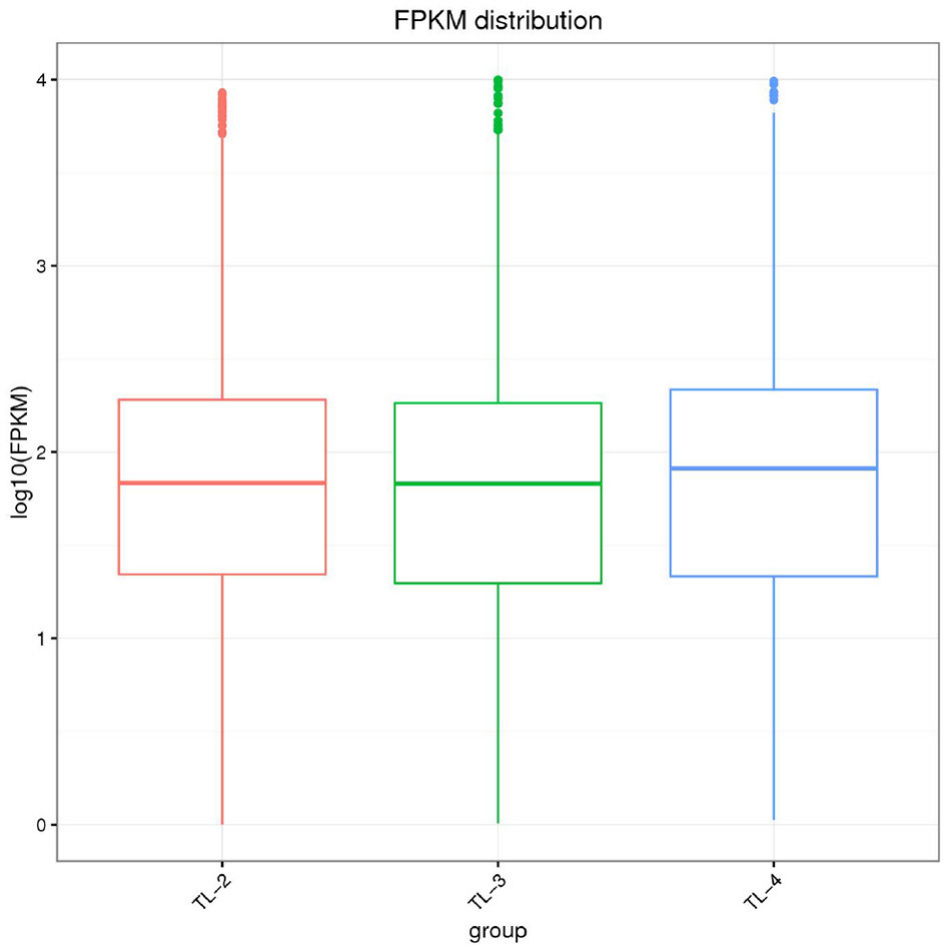
Statistical map of gene expression value distribution of each sample.

**Figure 2:**
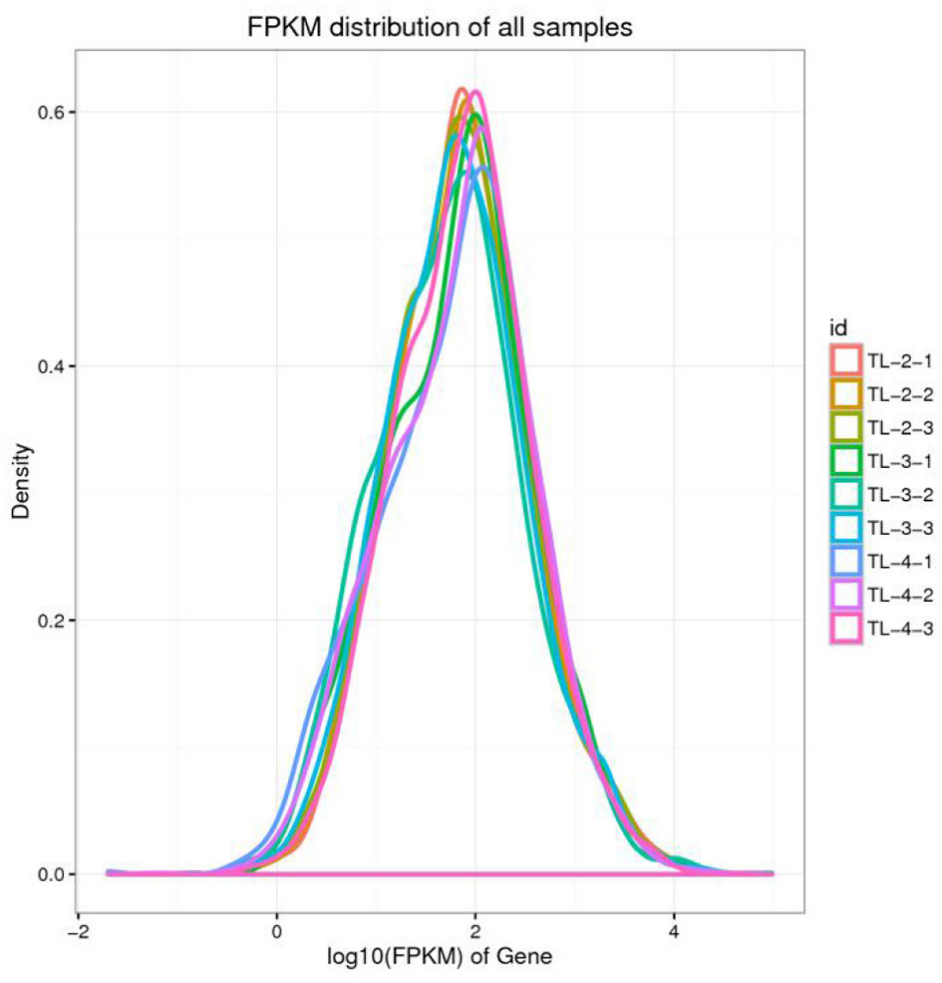
Density map of gene expression values of different samples. The abscissa is log10 (FPKM), and the ordinate is the density of genes.

The gene expression levels are calculated using the FPKM method. Due to the varying sequencing depths among different samples, the absolute expression levels of genes are amplified after the normalization of FPKM values. The intergroup comparison of gene expression levels is illustrated in the boxplot presented in Figure 1. Distribution plots of gene expression abundance are created by plotting the log10 (FPKM) values across different samples to compare the trends in expression changes (Figure 2). The expression density plots for each sample conform to a normal distribution, and the expression trends of biological replicates show consistency.

### 3.3 Indentification of differentially expressed genes

**Figure 3:**
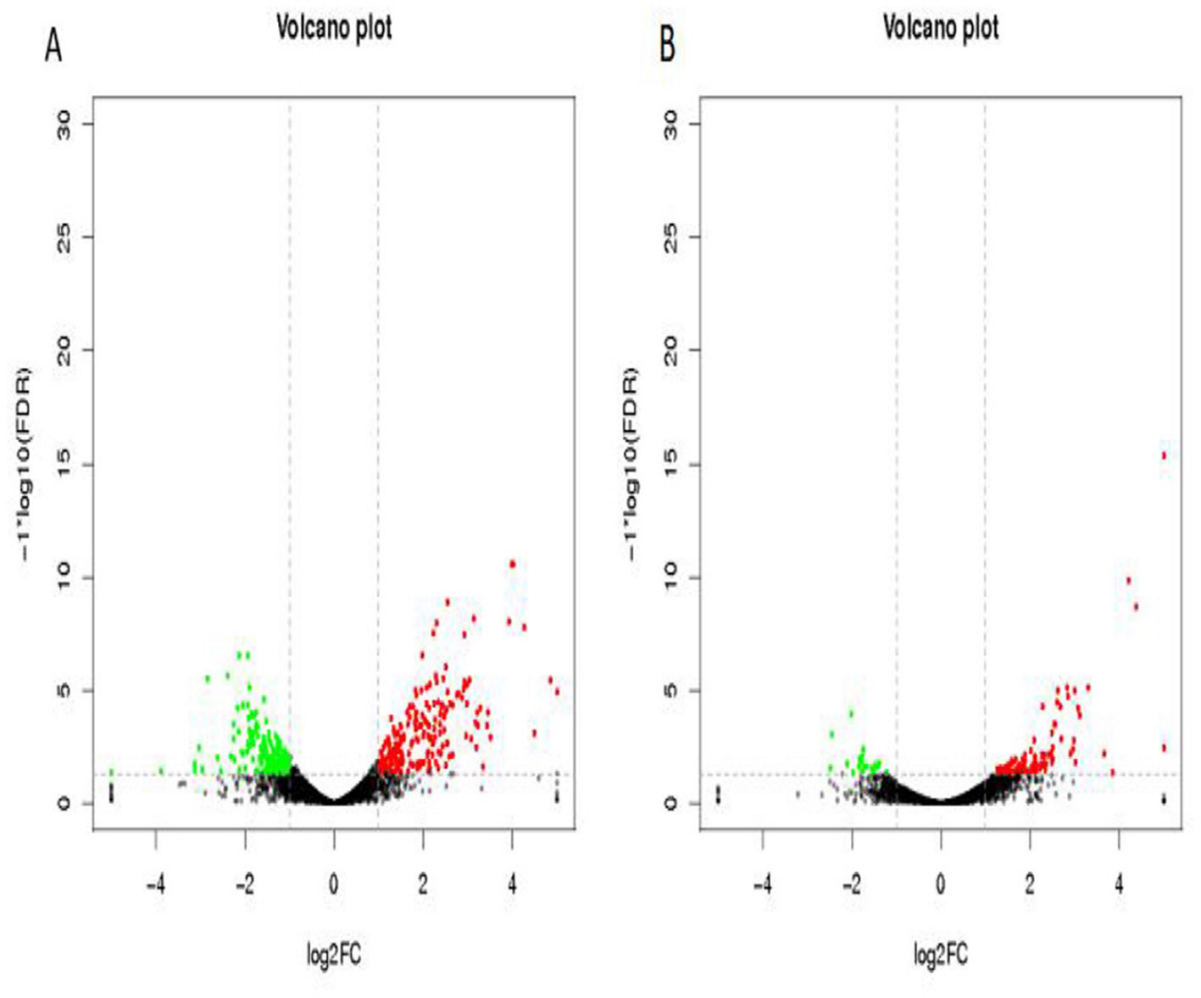
Volcano plots of differentially expressed genes. **(A)** TL-2 vs. TL-3 volcano plot, **(B)** TL-3 vs. TL-4 volcano plot. Black indicates genes with no significant changes, green indicates down-regulated genes, and red indicates up-regulated genes.

**Figure 4:**
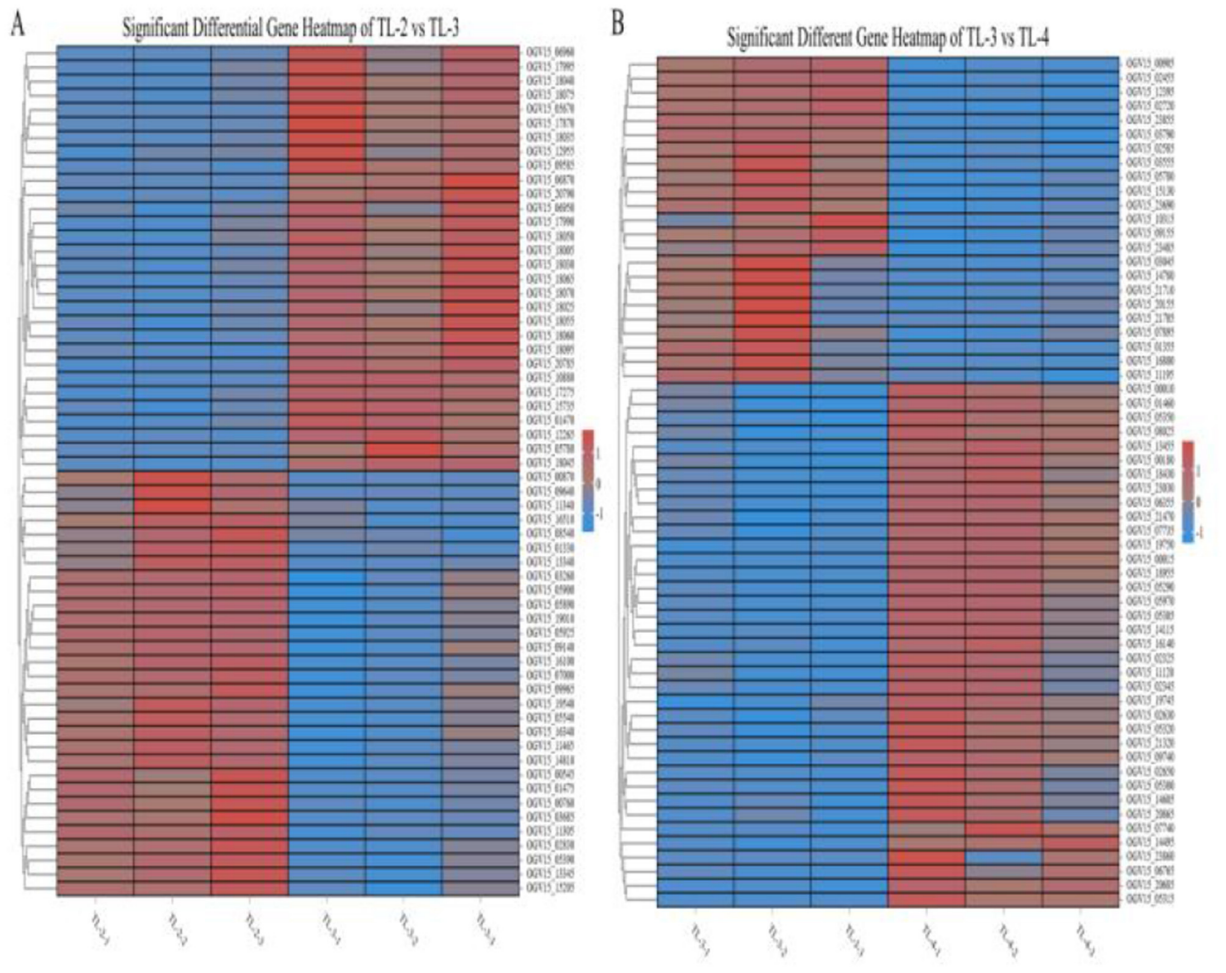
Heatmaps of partial differentially expressed genes. **(A)** Genes showing significant changes in the TL-2 vs. TL-3 comparison. **(B)** Genes showing significant changes in the TL-3 vs. TL-4 comparison.).

Differentially expressed genes were identified in edgeR software using the criteria |log2FC| > 1 and FDR < 0.05. The TL-2 vs. TL-3 comparison identified 415 DGEs (*P* < 0.05), including 201 upregulated and 214 down-regulated genes. As shown in Table 2, the DGEs showing the most significant up-regulation were *yiaG, gadA, rplW, yebG, cspI*, and *narG*, while the most significantly down-regulated DGEs were *yibA, gadE, argG, ydiE, artJ, htpG*, and *vapB*. The TL-3 vs. TL-4 comparison identified 125 DGEs (*P* < 0.05), including 102 upregulated and 23 downregulated genes. As shown in Table 3, the most significantly up-regulated genes were *dnaN, recF, recB, ycaQ, fimC, fimI*, and *fimA*, while the most significantly down-regulated genes were *yibA, gadE, argG, ydiE, artJ, htpG*, and *vapB*. Volcano plots (Figure 3) and heatmaps (Figure 4) illustrate the identified DEGs.

**Table 2:** Statistical results of TL-2 vs. TL-3 partial DEGs.

**id**	**TL-2_fpkm**	**TL-3_fpkm**	**Log2(FC)**	**Symbol**
OGV15_09585	836.1066667	1853.213333	1.148270056	yebG
OGV15_01125	717.4033333	2564.416667	1.837774336	yiaG
OGV15_01325	1446.636667	5720.43	1.983420973	gadA
OGV15_02330	108.9333333	602.7866667	2.468202023	rplW
OGV15_12735	41.60666667	363.3633333	3.12652623	narG
OGV15_11120	6.113333333	55.38333333	3.179420749	cspI
OGV15_00530	14.99666667	434.1033333	4.855324729	tetA
OGV15_22175	10.10666667	1.643333333	−2.620610202	rhaR
OGV15_20235	444.9866667	102	−2.125192956	nrdG
OGV15_05885	4008.32	1347.243333	−1.572987241	rpoE
OGV15_05895	3056.56	1406.956667	−1.119330992	rseB
OGV15_05890	8505.47	4023.836667	−1.079819214	rseA
OGV15_01330	5243.146667	2539.796667	−1.045719902	gadX
OGV15_00760	180.94	88.33333333	−1.034481518	rfaL

**Table 3:** Statistical results of TL-3 vs. TL-4 partial DEGs.

**id**	**TL-3_fpkm**	**TL-4_fpkm**	**Log2(FC)**	**Symbol**
OGV15_00010	105.4766667	312.95	1.569008292	dnaN
OGV15_00015	55.30333333	132.8	1.263816802	recF
OGV15_04790	45.94	168.9033333	1.878375038	recB
OGV15_14495	16.60666667	46.16	1.474880703	ycaQ
OGV15_19765	13.41	249.1033333	4.215363185	fimC
OGV15_19770	20.67666667	429.6233333	4.376996821	fimI
OGV15_19775	30.31666667	2494.053333	6.362237369	fimA
OGV15_00905	280.6333333	107.4933333	−1.384439193	yibA
OGV15_10315	71.09	12.62666667	−2.493172805	ydiE
OGV15_14780	434.36	79.22333333	−2.45489343	artJ
OGV15_01355	5371.47	1552.596667	−1.790633867	gadE
OGV15_23485	4802.973333	1441.856667	−1.736000044	vapB
OGV15_03045	414.5533333	125.61	−1.722606396	argG
OGV15_16800	2434.786667	828.69	−1.554890952	htpG

### 3.4 GO enrichment analysis of DEGs

**Figure 5:**
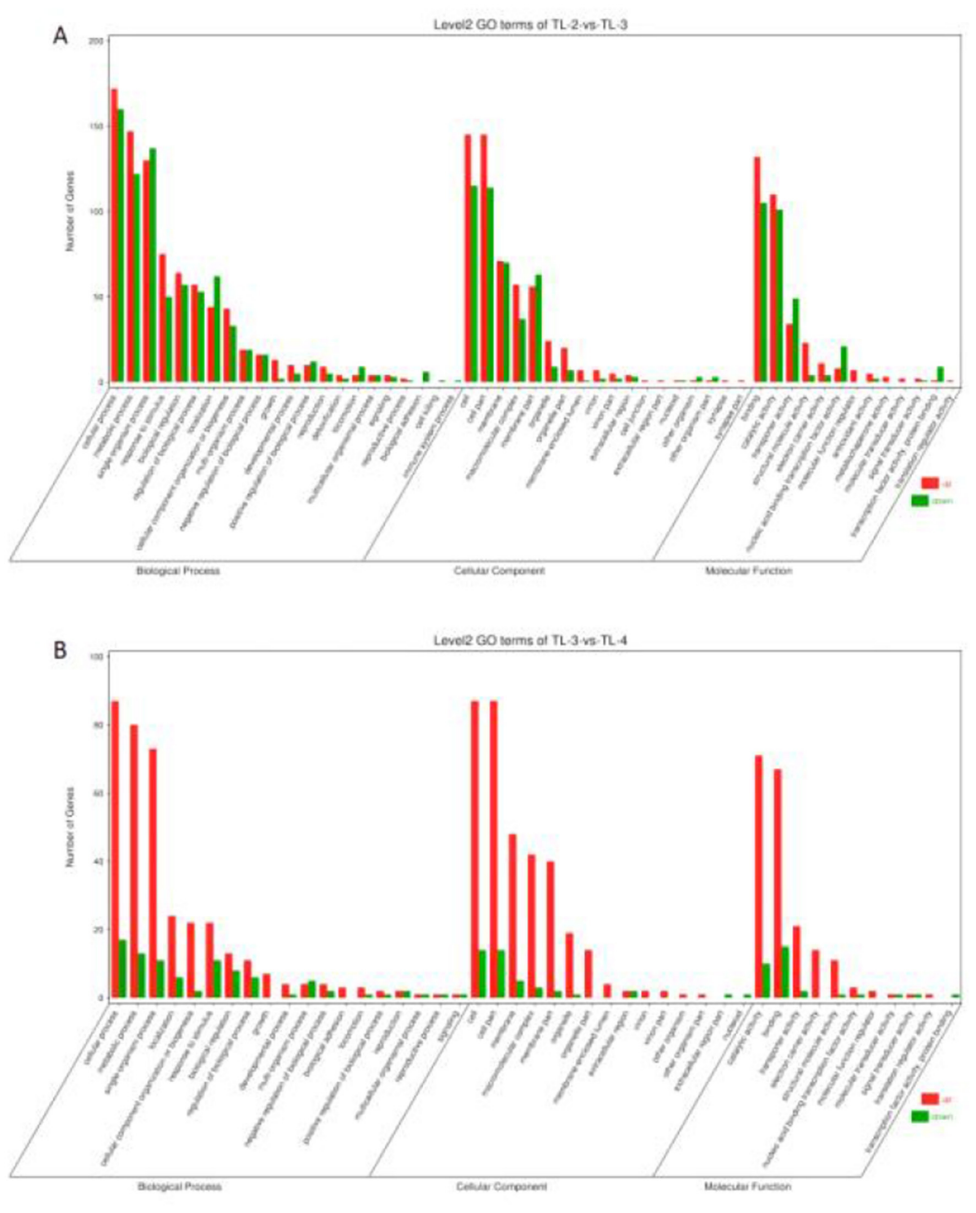
Gene Ontology enrichment analysis of differentially expressed genes. **(A)** TL-2 vs. TL-3 enrichment analysis. **(B)** TL-3 vs. TL-4 enrichment analysis.).

To compare the functions of the DEGs between the different groups, the up- and downregulated DGEs identified via edgeR were analyzed using GO, with a threshold value *P* < 0.05. The GO results were classified into cellular component (CC), molecular function (MF), and biological process (BP). The DEGs in the TL-2 vs. TL-3 comparison were classified into 13 MFs, 18 CCs, and 22 BPs, while those in the TL-3 vs. TL-4 comparison include 11 MFs, 15 CCs, and 19 BPs (Figures 5A, B). Categorizing the GO enrichment results in Level 2 GO terms showed that most of the DEGs were significantly enriched in metabolic, cellular, and single-organism processes in the BP category, cellular and cell membrane fractions in the CC category, and transporter activity, catalytic activity, and molecular binding in MFs.

### 3.5 KEGG enrichment analysis of DEGs

**Figure 6:**
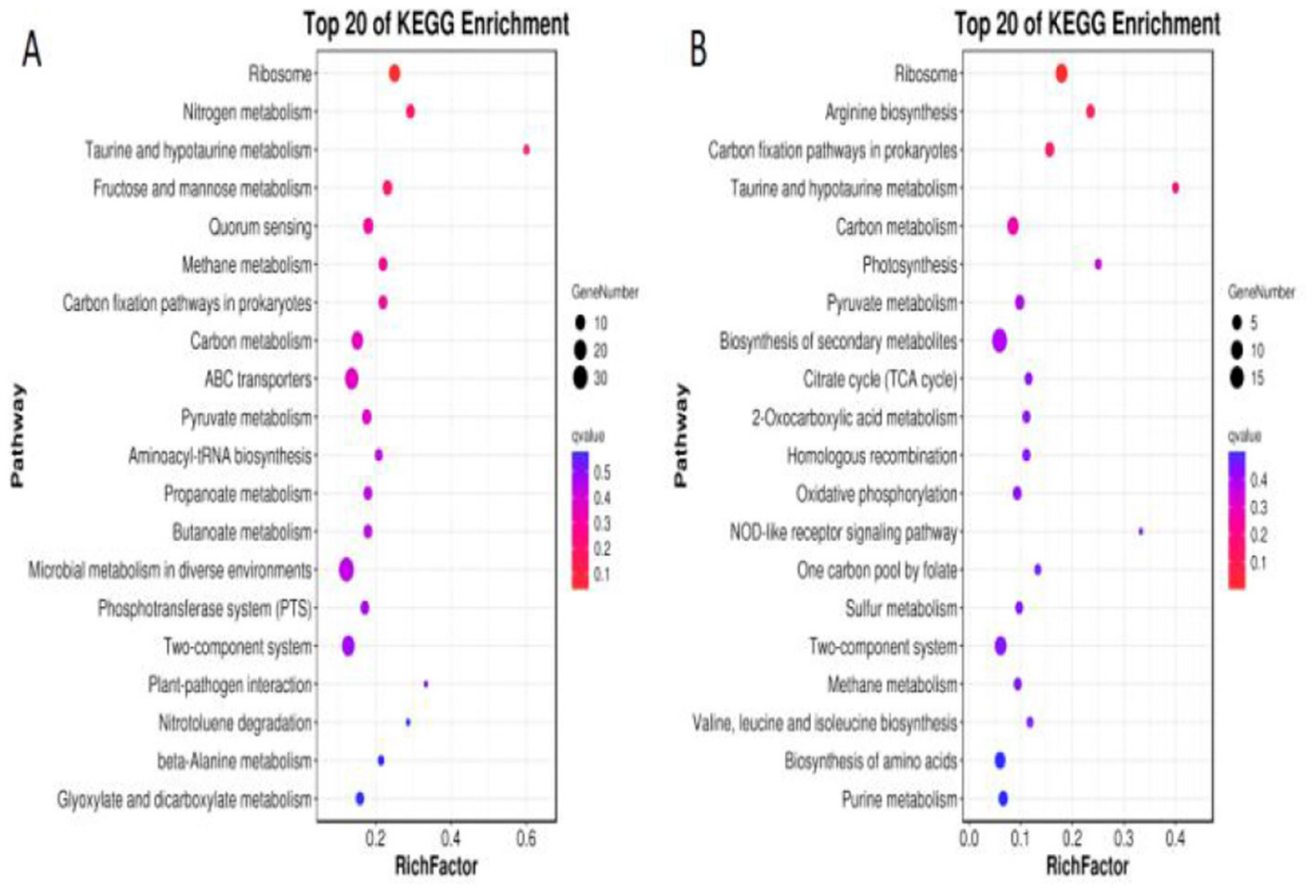
KEGG enrichment analysis of differentially expressed genes. **(A)** TL-2 vs. TL-3 comparison. **(B)** TL-3 vs. TL-4 comparison.).

To investigate which pathways may affect drug resistance in *E. coli*, all DEGs were further mapped using the KEGG database. The results are shown in Figure 6, with higher RichFactors indicating higher degrees of enrichment. The number of DEGs in the pathways is indicated by the size of the dots, with larger the dots representing greater numbers; dot colors indicate the *q*-value corrected by multiple hypothesis testing, with values ranging from 0 to 1, and the smaller the value, the more significant the enrichment. The results showed that the DEGs were mainly enriched in pathways associated with arginine, ribosomes, nitrogen metabolism, biosynthesis, and proline and arginine metabolism.

### 3.6 RT-qPCR verification

**Figure 7:**
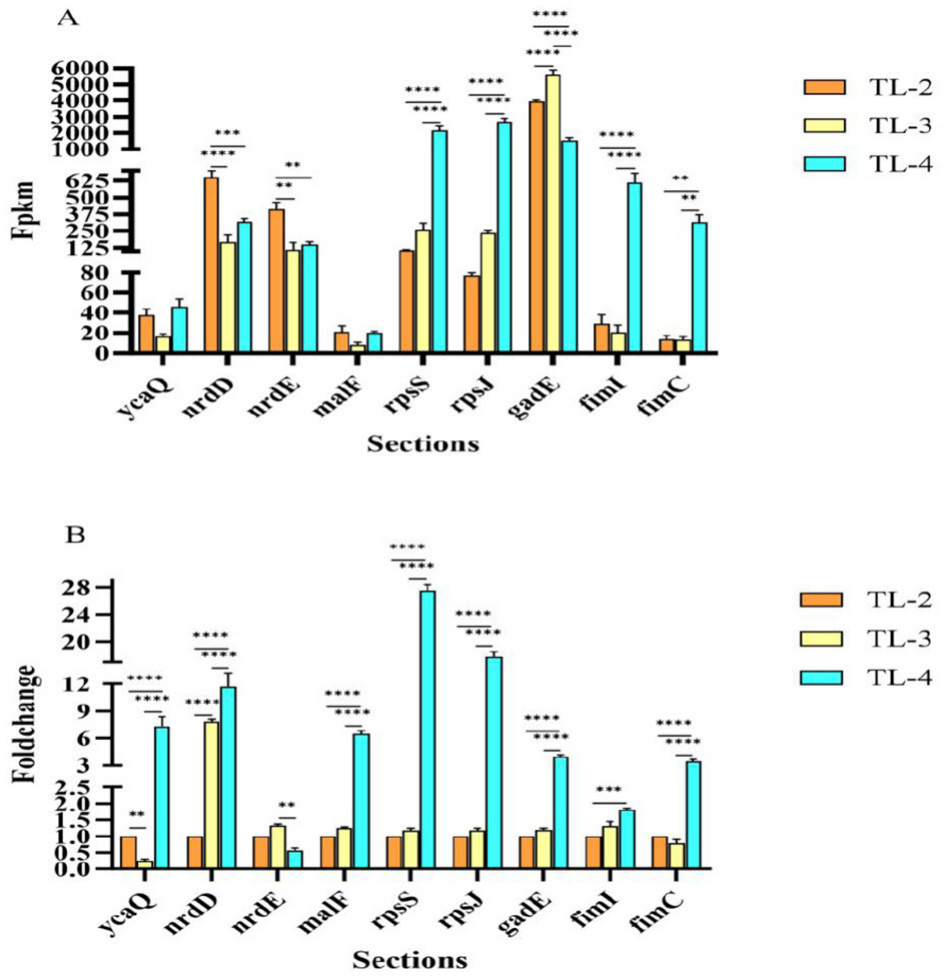
RT-qPCR verification of differentially expressed gene expression levels. **(A)** RT-qPCR verification of DGE FPKM values. **(B)** RT-qPCR verification of DGE fold changes). **, ***, ****represent significant differences at the 0.05 level, respectively.

To verify the reliability of the transcriptomic results, 9 DEGs were randomly selected for RT-qPCR verification (*ycaQ, nrdD, fimI, nrdE, gadE, rpsS, fimC, rpsJ*, and *maIF*). Measurement of the expression levels of these nine genes showed that the expression was consistent with the transcriptome sequencing results (Figures 7A, B), indicating that the transcriptome sequencing results were reliable.

## 4 Discussion

Neonatal calf diarrhea (NCD) caused by primary or secondary infections of *E. coli* has become one of the major diseases in the global cattle industry (25, 26). At present, the treatment of *E. coli* mainly depends on antibiotics. However, due to the extensive use of antibiotics in clinical treatment, *E. coli* is prone to drug resistance. Quinolones are antimicrobial agents used to treat pathogenic *E. coli* infections. In recent years, multidrug-resistant strains of *E. coli* have appeared, and *E. coli* has caused huge economic losses to the cattle industry worldwide. Ibrahim et al. discovered that 80.5% of the 41 *E. coli* strains were resistant to quinolones (27). *E. coli* is regarded as a reservoir of antibiotic resistance determinants. This bacterium readily develops antibiotic resistance and is capable of transferring antibiotic resistance factors to other pathogenic microbes in gastrointestinal tracts (28).

The OFX exert their actions by inhibiting bacterial nucleic acid synthesis through disrupting the enzymes topoisomerase IV and DNA gyrase, and by causing breakage of bacterial chromosomes (29–31). In this study, we found that the expression of some genes involved in DNA synthesis, such as *nrdD* and *nrdE*, decreased significantly after the addition of sub-inhibitory concentration of OFX, which was consistent with the antibacterial mechanism of OFX (32). In addition, the expression of *malF* gene was also significantly decreased. The *malF* gene is mainly involved in the decomposition and synthesis of sugar-containing compounds and the glycosylation of antibiotics in organisms (33). Therefore, it is speculated that OFX can also control the synthesis of membrane structures such as bacterial cell wall, cell membrane and biofilm by regulating the expression of *malF* gene, thereby inhibiting bacterial growth.

There are extensive studies on the regulation of bacterial virulence, acid resistance and biofilm formation by type I QS. Zhang et al. studied the mechanism of type I QS signal molecules to enhance the acid resistance of enterohemorrhagic *E. coli* 0157: H7. It was confirmed that the QS signal molecule AHL regulates the expression of a variety of acid resistant related genes by regulating the ribosome regulatory factor RMF to enhance its acid resistance, also the rmf gene also affects the survival ability of *E. coli* under various stress conditions (34). Studies on the regulation of bacterial virulence-related genes by type I QS have confirmed that the signaling molecules can enhance the stability of bacteria after colonization, both through the enhancement of biofilm formation ability to resist bacterial resistance to environmental stresses and enhance bacterial adhesion to colonized tissues, as well as regulating the expression of other adhesion factors, such as flagellum, bacteriophage, and so on (35). The regulation of bacterial biofilm by type I QS system not only affects the secretion of virulence factors, but is also very important for bacterial drug resistance. At present, there are relatively few studies on the regulation of bacterial resistance by type I QS. In this study, the addition of AHL signaling molecule C8 found that it had a regulatory effect on the resistance of multiple drugs in the tested strains, of which the regulatory effect on OFX was the most obvious. Transcriptome sequencing was performed on each treatment group at sub-inhibitory concentration, and functional annotation analysis was performed after obtaining DEGs. It was found that there may be two ways for AHL signaling molecules to regulate the resistance of the tested strains to OFX:

Firstly, it is involved in the regulation of bacterial SOS response, after adding AHL signaling molecules, the expression of some key genes of SOS response changed significantly, such as *recA, recN, recX*, etc. In addition, the expression of *ycaQ*, a newly discovered gene encoding glycosylase, also increased significantly in recent years, and the *ycaQ* protein can cut the interstrand DNA crosslinks (36). Therefore, it is hypothesized that the AHL signaling molecule can regulate the expression of the *ycaQ* gene, which cleaves the cross-links between OFX and DNA, and works in conjunction with the SOS response to protect the bacterial DNA and thus increase resistance to OFX.

Secondly, it is involved in the regulation of the bacterial CpxAR two-component system. The CpxAR two-component system is an important regulatory system in *E. coli*, which can regulate the formation of bacterial biofilms, the expression of multidrug efflux pump genes and so on, so it has a regulatory effect on the resistance of *E. coli*. The CpxAR two-component system is mainly composed of CpxA, CpxR, and CpxP. CpxA is a histidine protein kinase located in the inner membrane of the cell, CpxR is a response protein located in the cytoplasm, and CpxP is a response regulatory protein located in the periplasm (37). In this study, we found that after the addition of AHL signaling molecules, the expression of CpxP genes differed significantly, and the fimbria adhesin genes *fimA, fimC*, and *fimL* were significantly up-regulated, and some of the ABC efflux pump-related genes and the acid-tolerant gene, gadE, were also differentially expressed. Fimbria adhesin are an important virulence factor and are required for biofilm formation, and deletion of the *fim* gene significantly reduces the ability of the bacterial biofilm to form (38). The *gadE* gene belongs to the regulatory protein of the LuxR family. Schwan et al. have shown that the *gadE* gene may indirectly inhibit the transcription of the *fim* gene, thereby affecting the formation of biofilm (39). In this study, we found that the expression of *gadE* gene decreased significantly after the addition of AHL signaling molecules, while at the same time the expression of fimbria adhesin genes fimA, fimC, and fimL was significantly up-regulated, which is in line with the results of Schwan WR et al. Based on the above research results, it is speculated that AHL signaling molecules can affect the resistance of the tested bacteria to OFX by regulating the CpxAR two-component system.

Biofilms are highly resistant to immune killing and removal, as well as antibiotic treatment. Because biofilms have certain physical and chemical effects that make the internal bacteria less susceptible to antibiotics and thus more resistant to antibiotics (40, 41). After regulating the expression of fimbriae adhesin genes, the ability of biofilm formation can be enhanced, and the drug resistance of bacteria in biofilm can be greatly improved. In addition, studies have confirmed that the type I QS system can affect the bacterial multidrug resistance (MDR) system, and the QS and MDR regulatory systems may share signal molecule regulatory pathways (42). The multidrug efflux pumps used in the MDR regulatory system can simultaneously efflux QS signals. The mechanism of these efflux pumps to output drug molecules is similar to that of QS signaling molecules. High concentrations of antibiotics can induce more efflux pump genes to be overexpressed, and these molecules are recognized by *TetR* family regulators, such as the classical ABC efflux pump system in *E. coli*. The QS signal may have antibacterial activity, and antibiotics also play the role of signal molecules. The antibiotic efflux pump is also the output of the QS signal. Therefore, there may be a certain evolutionary relationship and biological correlation between QS and MDR regulatory systems (43).

## 5 Conclusion

In this study, we found that the AHL signaling molecule C8 enhanced the drug resistance of the strains by adding 200 mg/L (1 × 10^−3^ mol/L) of AHL signaling molecule C8 to the subject strains, with a significant increase in resistance to OFX. The DEGs were obtained by transcriptome sequencing. Through the functional annotation analysis of the DEGs, it was speculated that there may be two ways for AHL signaling molecules to regulate the resistance of the tested strains to OFX: Firstly, it is involved in regulating the SOS response of bacteria, as well as regulating the expression of *ycaQ*, a gene encoding a glycosylase, to protect bacterial DNA and thereby increase resistance to OFX. Secondly, it is involved in the regulation of the bacterial CpxAR two-component system, which regulates the formation of bacterial biofilm and thus affects the resistance of the tested strain to OFX.

## Data Availability

The datasets presented in this study can be found in online repositories. The names of the repository/repositories and accession number(s) can be found below: https://www.ncbi.nlm.nih.gov/, PRJNA1163313.
