# Supplementary material for: Regulation of ofloxacin resistance in Escherichia coli strains causing calf diarrhea by quorum-sensing acyl-homoserine lactone signaling molecules
Source: Front Vet Sci. 2025 Feb 5;12:1540132. doi: 10.3389/fvets.2025.1540132 (PMC11837865; doi:10.3389/fvets.2025.1540132)
Supplement: Supplementary file 1 [file Table_1.docx]

Table S1 Validation of gene primer sequences

| Primer name | Primer sequence (5´→3´) |
| --- | --- |
| q16S-F | GAGCAAGCGGACCTCATA |
| q16S-R | ATTCACCGTGGCATTCTG |
| fimI-F | GCCAGGGATAGCCACAAA |
| fimI-R | GCGAAGTAGAGCCTGAGTAAA |
| fimC-F | GGGAAAGTTTATTCTGGATG |
| fimC-R | CAGTTTAATGCGGCTGAT |
| rpsJ-F | TGCCGACACGCAAAGAGC |
| rpsJ-R | AGACGCATCAGAGCATCAA |
| rpsS-F | AAGCGGAGACAAGAAGCC |
| rpsS-R | ATTATGGACAGCGATGGT |
| ycaQ-F | GTCCGCAACCAAATGTATC |
| ycaQ-R | CGTCACGCAACTGCTCCA |
| gadE-F | AGGCAATAAACCCTTCAA |
| gadE-R | TCGGCATCTAATTTCTCC |
| nrdD-F | GAAAGAAGAAACGGGTTATGG |
| nrdD-R | GAGGTGGAAACTGTTGGTGTAG |
| nrdE-F | CCGTTGACGCCTTTATTG |
| nrdE-R | TTCGTTGACCAGCCAGTT |
| malF-F | TTTCGCCAATCGTAAAGC |
| malF-R | CAGACCAGAGGGAAGAGGAC |
